# Racial differences in prostate inflammation: results from the REDUCE study

**DOI:** 10.18632/oncotarget.10690

**Published:** 2016-07-18

**Authors:** Adriana C. Vidal, Zinan Chen, Lauren E. Howard, Daniel M. Moreira, Ramiro Castro-Santamaria, Gerald L. Andriole, Emanuela Taioli, Jay H. Fowke, Beatrice Knudsen, Charles G. Drake, J. Curtis Nickel, Stephen J. Freedland

**Affiliations:** ^1^ Department of Surgery, Division of Urology, Cedars Sinai Medical Center, Los Angeles, CA, USA; ^2^ Department of Biostatistics and Bioinformatics, Duke University School of Medicine, Durham, NC, USA; ^3^ Department of Urology, Mayo Clinic, Rochester, MN, USA; ^4^ GlaxoSmithKline Inc., R&D, King of Prussia, PA, USA; ^5^ Washington University School of Medicine in St. Louis, St. Louis, MO, USA; ^6^ Institute for Translational Epidemiology, Icahn School of Medicine at Mount Sinai, New York, NY, USA; ^7^ Department of Medicine and Urologic Surgery, Vanderbilt University Medical Center, TN, USA; ^8^ Department of Medicine, Cedars-Sinai Biobank, Cedars Sinai Medical Center, Los Angeles, CA, USA; ^9^ Johns Hopkins Sidney Kimmel Comprehensive Cancer Center, and the Brady Urology Institute, John Hopkins University, Baltimore, MD, USA; ^10^ Department of Urology, Queen's University, Kingston, ON, Canada

**Keywords:** prostate biopsy, inflammation, race, prostate cancer, acute inflammation

## Abstract

Prostate cancer (PC) risk differs between races, and we previously showed prostate inflammation in benign prostate tissue was linked with a lower future PC risk. However, whether prostate tissue inflammation varies by race is unknown. We analyzed baseline acute and chronic prostate inflammation by race in REDUCE, a 4-year, multicenter, placebo-controlled study where all men had a negative prostate biopsy prior to enrollment. We included 7,982 men with standardized central pathology review to determine the presence or absence of chronic or acute inflammation in baseline prostate biopsy tissue. Logistic regression was used to compare prostate inflammation by race, adjusting for confounders. Of 7,982 men, 7,271 were white (91.1%), 180 (2.3%) black, 131 (1.6%) Asian, 319 (4.0%) Hispanic and 81 (1%) unknown. A total of 78% had chronic and 15% had acute inflammation. On multivariable analysis relative to white men, black men were less likely (OR = 0.65, 95%CI: 0.41-1.03, *p* = 0.07) and Asian men more likely to have acute inflammation (OR = 1.74, 95%CI: 1.14-2.65, *p* = 0.001). Hispanic men had similar levels of acute inflammation as white men. Chronic inflammation did not significantly differ across races. We identified racial differences in acute inflammation, particularly in Asian men, in benign prostate tissue that inversely mirrored population-level data on PC race disparity. As we showed in REDUCE that acute inflammation is linked with lower future PC risk, if validated in future studies, these data suggest racial differences in prostatic acute inflammation may contribute in part to race differences in PC risk, especially among Asian men.

## INTRODUCTION

While it has been postulated inflammation drives prostate cancer (PC) development [1], the link between inflammation and PC risk appears to be complex. Indeed some studies suggest inflammation may even be associated with lower PC risk [2, 3]. Moreover, other data suggest there are differences between races in the degree of inflammatory gene expression within the tumors [4, 5].

The REDUCE study provides a unique opportunity to evaluate the association between inflammation and future PC risk. REDUCE was a multinational randomized clinical trial designed to compare the effect of dutasteride on PC diagnosis among men with a negative pre-study biopsy and elevated PSA [6]. This biopsy was centrally reviewed by a single pathologist who graded it systematically for inflammation. Analyzing negative biopsies from 6,238 men in REDUCE who had a subsequent biopsy, we previously found acute and chronic inflammation was present, respectively, in 15% and 77% of negative biopsies [2]. The presence of inflammation, either acute or chronic, was linked with a 35-40% lower PC risk on a biopsy taken 2 years later. However, on the 4-yr biopsy only acute inflammation was associated with reduced PC risk. Similar results were found in a Finnish study of 293 men with a negative biopsy, with histological inflammation associated with decreased PC risk at follow-up [3]. Collectively these studies suggest *acute* inflammation in a benign biopsy may portend a lower future PC risk. In contrast, a study of 400 men (84% white) in the placebo arm of the PC Prevention Trial (PCPT) who underwent end of study biopsy, men with PC on biopsy, especially high-grade PC, were more likely to have inflammation (typically chronic) in the benign cores from the same biopsy *vs*. men without PC [7]. Likewise, we previously found predominantly chronic inflammation in PC tissue correlated with worse outcomes (recurrence after surgery) [8]. This suggests the co-occurrence of chronic inflammation and PC correlates with more aggressive PC. Whether these disparate results reflect differences in acute *vs*. chronic inflammation or differences in examining benign prostate tissue predicting future PC risk *vs*. looking at PC tissue is not clear.

Some studies suggest there may be race differences in inflammation. Data from NHANES from 1999-2002 found healthy black men have higher levels of circulating inflammatory markers, such as C-reactive protein (CRP) and fibrinogen, *vs*. white men [9]. Another study found Black men had significantly higher prostate tumor expression of IL1β, IL6, and IL8 compared to white men [4]. One prior small (*n* = 238) study did find inflammation was equally common in radical prostatectomy tissue of white *vs*. black men [10]. However, that study did not discriminate between acute and chronic inflammation, and since all men had PC, the potential influence of the tumor on inflammation levels could not be addressed. Importantly, data of prostate tissue inflammation are limited on men of races beyond black and white.

Given prostate tissue inflammation in REDUCE is associated with lower future PC risk, and PC incidence and mortality rates vary by race (higher among black men and lower among Asian men *vs*. whites [11]), we determined whether prostate inflammation varied by race by analyzing prostate inflammation patterns in negative biopsies in REDUCE, where men were recruited globally. We hypothesized prostate inflammation would vary by race with black men having less inflammation (suggesting higher PC risk) and Asian men would more inflammation (suggesting lower PC risk) *versus* white men.

## RESULTS

### Study subjects demographics

In our study cohort (*n* = 7,982), 7,271 (91%) were white, 180 (2%) were black, 131 (2%) were Asian, 319 (4%) were Hispanic, and 81 (1%) were men of unknown race. Race was significantly associated with geographic region (*p* < 0.001), abnormal DRE (*p* < 0.001), PSA (*p* = 0.018), prostate volume (*p* < 0.001), and aspirin and/or NSAID use (*p* < 0.001) (Table 1). Race was also significantly associated with the presence of acute inflammation (*p* = 0.015), but not with the presence of chronic inflammation (*p* = 0.112) in the pre-study negative biopsy (Table 1).

**Table 1:** Baseline Patient Characteristics by Race (*N* = 7901)

	Race				
	White	Black	Asian	Hispanic	*P*-value
**No. patients** (%)	7271 (92%)	180 (2%)	131 (2%)	319 (4%)	-
**Age** (year), Mean (SD)	62.8 (6)	62.9 (6.2)	62.3 (6.7)	62.2 (6.2)	0.37^1^
**Region** (%)					<0.001^2^
North America	1783 (24%)	135 (75%)	57 (44%)	76 (24%)	
Europe	4787 (66%)	12 (7%)	7 (5%)	2 (1%)	
Other	701 (10%)	33 (18%)	67 (51%)	241 (75%)	
**DRE** (%)					0.001^2^
Normal	7016 (97%)	165 (92%)	127 (97%)	299 (94%)	
Abnormal	255 (3%)	15 (8%)	4 (3%)	20 (6%)	
**PSA** (ng/mL), M(Q1,Q3)	5.7 (4.4, 7.3)	5.3 (4.2, 7.0)	5.5 (4.1, 7.3)	5.2 (4.1, 7.3)	0.018^3^
**PV** (cm^3^), M(Q1,Q3)	43.4 (32.9, 56.3)	43.7 (34.2, 62.1)	34.2 (26.3, 46.5)	47.4 (35.9, 61.5)	<0.001^3^
**Smoking** (%)					0.076^2^
Never	3323 (46%)	91 (50%)	49 (37%)	153 (48%)	
Former	2860 (39%)	59 (33%)	52 (40%)	123 (39%)	
Current	1088 (15%)	30 (17%)	30 (23%)	43 (13%)	
**Aspirin/NSAID use** (%)					<0.001^2^
No	5192 (71%)	114 (63%)	106 (81%)	269 (84%)	
Yes	2079 (29%)	66 (37%)	25 (19%)	50 (16%)	
**Acute Inflammation** (%)	1113 (15%)	24 (13%)	34 (26%)	45 (14%)	0.007^2^
**Chronic Inflammation** (%)	5616 (77%)	148 (82%)	105 (80%)	259 (81%)	0.133^2^
**No Inflammation** (%)	1595 (22%)	32 (18%)	25 (19%)	57 (18%)	0.162^2^
**Biopsy Results** (%)					<0.001^2^
No on-study biopsies	1202 (17%)	52 (30%)	23 (18%)	55 (18%)	
1 negative on-study biopsy	734 (11%)	26 (15%)	15 (12%)	29 (9%)	
2 negative on-study biopsies	3736 (53%)	62 (36%)	76 (59%)	171 (55%)	
Cancer detected on-study	1367 (19%)	33 (19%)	15 (11%)	55 (18%)	

Abbreviations: DRE, digital rectal examination; PSA, prostate-specific antigen; PV, prostate volume; SD, standard deviation; M, Median; Q1, 25^th^ percentile; Q3, 75^th^ percentile.

^1^*P*-value calculated using Student ANOVA; ^2^ *P*-value calculated using Chi-squared test; ^3^*P*-value calculated using Kruskal-Wallis test.

### Inflammation and race

On univariable analysis, with white race as the reference, Asian men were more likely to have acute inflammation (OR = 1.94, 95%CI: 1.31-2.88, *p* = 0.001) (Table 2). No other races were significantly associated with acute inflammation. After adjusting for demographic and clinical characteristics, black race was associated with a suggestively lower likelihood of having baseline acute inflammation (OR = 0.65, 95%CI: 0.41-1.03, *p* = 0.065) while the likelihood of having acute prostate inflammation remained significantly higher in Asians *vs*. white men (OR = 1.74, 95%CI: 1.14-2.65, *p* = 0.010). Hispanic race/ethnicity remained not significantly associated with acute inflammation on multivariable analysis. Given the differences in results between univariable and multivariable analyses between black men and acute inflammation risk, we examined which factor had the greatest influence on altering the odds ratio by adding each variable to the multivariable model one at a time. When this was done, we found that region and prostate volume had the greatest effect on altering the OR between univariable and multivariable analyses.

**Table 2:** Association between Baseline Acute Inflammation and Race

		Univariable		Multivariable*	
Patient Race	No. (%)	OR (95% CI)	*P*-Value	OR (95% CI)	*P*-value
White	7271 (92.0)	*Ref.*	*Ref.*	*Ref.*	*Ref.*
Black	180 (2.3)	0.85 (0.55, 1.31)	0.467	0.65 (0.41, 1.03)	0.065
Asian	131 (1.7)	1.94 (1.31, 2.88)	0.001	1.74 (1.14, 2.65)	0.010
Hispanic	319 (4.0)	0.91 (0.66, 1.25)	0.560	1.04 (0.73, 1.49)	0.826

Abbreviations: 95% CI, 95% confidence interval; OR odds ratio.

*Adjusted for baseline age, race, region, DRE (digital rectal examination), prostate volume, PSA (prostate-specific antigen),smoking, aspirin/NSAID use, and biopsy results.

There were no significant associations between prostate chronic inflammation and race in either univariable or multivariable analysis (Table 3).

**Table 3:** Association between Baseline Chronic Inflammation and Race

		Univariable		Multivariable*	
Patient Race	No. (%)	OR (95% CI)	*P*-Value	OR (95% CI)	*P*-value
White	7271 (92.0)	*Ref.*	*Ref.*	*Ref.*	*Ref.*
Black	180 (2.3)	1.36 (0.93, 2.01)	0.116	1.11 (0.74, 1.67)	0.611
Asian	131 (1.7)	1.19 (0.77, 1.83)	0.431	1.01 (0.65, 1.59)	0.949
Hispanic	319 (4.0)	1.27 (0.96, 1.69)	0.099	1.04 (0.76, 1.42)	0.822

Abbreviations: 95% CI, 95% confidence interval; OR odds ratio.

*Adjusted for baseline age, race, region, DRE (digital rectal examination), prostate volume, PSA (prostate-specific antigen),smoking, aspirin/NSAID use, and biopsy results.

### Sensitivity analyses

In sensitivity analysis, we were concerned as region and race were closely related and including regions with limited racial diversity may affect the results. Specifically, men enrolled in Europe were rarely black (*n* = 12, 0.2%) or Asian (*n* = 7, 0.1%). Therefore, we repeated our analysis excluding men from Europe and results were little changed (Supplementary Table 1). Similarly, when not adjusting for prostate cancer diagnosis, results were little changed (data not shown). When stratifying analyses by NSAID and/or aspirin use, we found acute inflammation remained positively associated with Asian race (OR = 1.82 95%CI: 1.13-2.93, *p* = 0.014), and inversely associated with black race, (OR = 0.42, 95%CI: 0.22-0.82, *p* = 0.012) among non-users only. While there were no significant associations between any race and acute inflammation among NSAID and/or aspirin users (Supplementary Table 2), none of the interactions between race and NSAID and/or aspirin use were significant (all p-interaction≥0.072), suggesting the associations between race and inflammation were statistically the same regardless of NSAID and/or aspirin use.

## DISCUSSION

Age, race and family history are well-established PC risk factors. PC rates in Asians are lower than in whites and Hispanics, while black men are disproportionally affected by PC [12]. The reasons for these disparities are unclear. In trying to understand a mechanistic underpinning of race disparities, we note some data suggesting racial differences in inflammation [9, 13], as well as data linking inflammation and PC risk, albeit with conflicting results [2, 3, 7, 14, 15]. However, few studies analyzed the presence and type of inflammatory cells (i.e. acute *vs*. chronic) within the prostate by race. To test this, we investigated the relationship between prostate inflammation, both chronic and acute, and race in REDUCE, where all men had a negative pre-study biopsy and wherein we previously showed inflammation was related to lower future PC risk [2]. As we hypothesized, compared to white men, black men were less likely and Asian men more likely to have acute prostate inflammation in their negative biopsies, though associations for black men were not statistically significant. No associations were found between chronic prostate inflammation and race. These findings suggest racial differences in acute prostate inflammation may explain, in part, racial differences in PC risk, especially among Asian men.

Few studies to date analyzed prostate inflammation as a function of race. A small study (*n* = 210 of which 49 were black) analyzed radical prostatectomy tissue and reported black men had similar rates of inflammation as white men [10]. However, results were not stratified by acute *vs*. chronic inflammation. Given chronic inflammation is more common than acute, these results were most likely driven by chronic inflammation. A recent prostate cancer case-control study (*n* = 345 white, *n* = 229 black men) examined glandular and stromal inflammation by race [16]. Consistent with the prior study, they found no overall difference in rates of histological inflammation. However, black men had 32% lower rates of glandular inflammation. Whether glandular inflammation is the same as acute inflammation is not exactly clear as one is defined by cellular compartment (i.e. glandular) whereas acute is defined by the type of cells present. Nonetheless, this study did suggest some racial differences in prostatic inflammation levels. Another small study by Zlotta et al. (*n* = 320 of which 100 were Asian) analyzed prostate inflammation on autopsies of Asian and Caucasian men with no known history of PC [17] and also found no differences in inflammation by race. In this study, the authors examined acute and chronic inflammation separately and found no association with either type of inflammation. Our results are in-part in line with these data. Specifically, we also found that on negative pre-study prostate biopsies, there were no differences in chronic inflammation by race. However, we found acute prostate inflammation was more prevalent in Asians and suggestively less prevalent in black men compared to white men. Thus, while all 3 studies agree on the lack of association between race and chronic inflammation, there is disagreement on acute inflammation. In part this may stem from statistical power. Acute inflammation was present in only 15% of biopsies in our study (12% in the Zlotta et al paper). Thus, with few events, it may be tough to detect associations in a study with a few hundred men. However, as our study included 7,982 men of which 131 were Asian, we had increased power to observe differences in an uncommon event such as acute inflammation between races. However, despite being a slightly larger study, our overall numbers of non-white men was limited. As such, whether the limited number of black men affected our ability to detect a significant association between acute inflammation with black race or whether this truly reflects no association requires further study. Alternatively, study population differences may account for the different results. Our study included a more homogenous group - all with an elevated PSA and a negative prostate biopsy who were healthy enough to enroll on a phase III PC prevention study. The Zlotta et al, study included men who died including a large percentage who died of cancers (other than prostate) - 56% of the Asian men. Finally, there were differences in statistical approaches. The Zlotta et al, study did not perform multivariable analysis despite clear differences between Asians and Caucasians - 6 year difference in age, a 20% difference in prostate size, and a big difference in history of non-PC (59% in the Asians *vs*. 12% in Caucasians). Whether adjusting for these differences would have affected the results is unknown.

While the role of inflammation in prostate tissue is controversial, our previous data from the same dataset showed both acute and chronic inflammation in pre-study negative biopsy was associated with reduced PC risk in repeat 2-yr biopsies [2]. However, only acute inflammation predicted lower PC risk at the 4-yr biopsy. Given differences in inflammation by race were only in acute inflammation, this raises the hypothesis that racial differences in inflammation may in part be responsible for PC racial disparities. If our results are validated in future studies, additional work is needed to determine whether inflammatory cell infiltrate difference results from host differences in genetics, lifestyle, or prostate microenvironment [18]. Perhaps more interestingly, the suggestion that acute inflammation may be protective against the future development of PC is intriguing and requires further follow up. In lung cancer, neutrophilic infiltration (acute inflammation) has recently been shown to include both N1 (protective) and N2 (pro-carcinogenic) phenotypes [19]. Follow-up studies designed to comprehensively profile the phenotypic characteristics of prostatic acute inflammation are under way.

Our study had key strengths: large samples of white subjects, centrally and systematically read pathology, and data available on key confounding variables including PSA, prostate size, smoking, and aspirin and/or NSAID use. However, though our study was larger than prior studies, the number of black and Asian men was modest and only slightly larger than prior studies. While we defined inflammation as obtained from central pathology as acute or chronic, a more detailed analysis of the exact cells that exist within the prostate may yield greater insight into the association between race, inflammation, and PC biology. As noted, a key strength of this study is that inflammation was systematically graded by a single pathologist and the uniform enrollment criteria.

## CONCLUSIONS

We found that in men with negative prostate biopsies and an elevated PSA, black men had suggestively less acute inflammation while Asian men had significantly more acute inflammation with no race differences in chronic inflammation. If confirmed in future studies, these results suggest racial differences in prostatic inflammation, especially in Asian men, may in part be involved in explaining racial differences in PC risk.

## MATERIALS AND METHODS

### Study population

REDUCE was a 4-year, multicenter, randomized, double-blind, placebo-controlled study. While study details are described elsewhere, they are not relevant to this analysis in that we only used baseline data [6, 20]. Eligibility requirements were age 50-75 years old, serum PSA 2.5-10.0 ng/ml if 50-60 years or 3.0-10.0 ng/ml if > 60 years, and a single, negative prostate biopsy (6-12 cores) within 6 months prior of enrollment and independent of the study. Men with past history of PC, prostate surgery, prostate volume > 80 ml, or IPSS > 25 or > 20 on alpha-blockers were excluded. Of 8,122 subjects in the efficacy population, we excluded men with missing baseline data on race (*n* = 1), PSA (*n* = 18), DRE (*n* = 15), prostate volume (*n* = 100), or smoking status (*n* = 6). A total of 7,982 subjects met the inclusion criteria.

Each core was centrally read by a single pathologist and coded as present or absent for acute and chronic prostate inflammation. A patient was considered to have inflammation if any core was positive for inflammation. Chronic inflammation consisted mainly of lymphocytes and a variable number of plasma cells and macrophages. Acute inflammation consisted of a neutrophilic infiltrate [2]. Thus, as acute and chronic inflammation are not mutually exclusive, a patient could have no inflammation, acute only, chronic only, or both. As the pathophysiology of acute and chronic inflammation likely differ, they were analyzed as separate and independent conditions. Race was self-reported as “black”, “white”, “Hispanic”, “Asian”, or “other”.

### Statistical analysis

The association between baseline acute and chronic inflammation and baseline clinical characteristics was examined using student *t*-test, Wilcoxon rank-sum or χ^2^ for continuous normal, continuous non-normal and categorical variables, respectively. Logistic regression was used to test the association between race (primary predictor variable) and presence or absence of baseline acute and chronic inflammation (primary outcome variables). Multivariable analyses were adjusted for baseline age (continuous), PSA (continuous), DRE (abnormal *vs*. normal), smoking status (non-smoker, former, current), aspirin and/or NSAID use (yes *vs*. no), and geographic region (North America, Europe, other). As all men in this study had an elevated PSA and given that inflammation as well as prostate cancer and prostatic enlargement are known causes of an elevated PSA, we further adjusted for prostate volume (continuous), and a 4-tiered categorical variable of whether over the course of the 4-year REDUCE study, the subjects had 1 negative prostate biopsy *vs*. 2+ negative biopsies *vs*. no on-study biopsy, or found to have prostate cancer on follow-up biopsy. Sensitivity analyses were conducted excluding men from Europe, as there were few black and Asian men (race/ethnicity was self-reported). We also conducted a subgroup analysis among NSAID and/or aspirin users and non-users. Also, we examined the effect of not adjusting for future prostate cancer diagnosis given this information was not available at the time of baseline. Statistical significance was defined as *p* < 0.05. All analysis was performed using Stata *v13.1.*

## SUPPLEMENTARY MATERIALS TABLES


