# Supplementary material for: Racial differences in prostate inflammation: results from the REDUCE study
Source: Oncotarget. 2016 Jul 18;8(42):71393–9. doi: 10.18632/oncotarget.10690 (PMC5641057; doi:10.18632/oncotarget.10690)
Supplement: Supplementary file 1 [file oncotarget-08-71393-s001.pdf]

Racial differences in prostate inflammation: results from the REDUCE study

Supplementary Material

Supplementary Table 1: Sensitivity analysis among none or mild prostatitis patients (N=5025)

		Acute Inflammation				Chronic Inflammation			
		Univariable		Multivariable*		Univariable		Multivariable*	
**Patient Race**	**Total No. (%)**	**OR (95% CI)**	**P-Value**	**OR (95% CI)**	**P-value**	**OR (95% CI)**	**P-value**	**OR (95% CI)**	**P-value**
White	4538 (90.3)	*Ref.*	*Ref.*	*Ref.*	*Ref.*	*Ref.*	*Ref.*	*Ref.*	*Ref.*
Black	128 (2.6)	0.75 (0.45, 1.26)	0.284	0.60 (0.35, 1.01)	0.055	1.27 (0.80, 2.03)	0.307	1.14 (0.70, 1.88)	0.593
Asian	116 (2.3)	1.49 (0.96, 2.31)	0.072	1.52 (0.96, 2.42)	0.074	1.13 (0.71, 1.81)	0.611	1.04(0.64, 1.69)	0.872
Hispanic	243 (4.8)	0.77 (0.53, 1.13)	0.180	0.90(0.59, 1.36)	0.610	1.05 (0.76, 1.44)	0.780	0.90 (0.63, 1.29)	0.570

Abbreviations: 95% CI, 95% confidence interval; OR odds ratio.
*Adjusted for baseline age, race, region, DRE (digital rectal examination), prostate volume, PSA (prostate-specific antigen), smoking, aspirin/NSAID use, and biopsy results.

Supplementary Table 2: Association between Baseline Inflammation and Race Stratified by NSAID use

		Acute Inflammation				Chronic Inflammation			
		Univariable		Multivariable*		Univariable		Multivariable*	
**Patient Race**	**Total No. (%)**	**OR (95% CI)**	**P-Value**	**OR (95% CI)**	**P-value**	**OR (95% CI)**	**P-value**	**OR (95% CI)**	**P-value**
**Non-NSAID Users**									
White	5192 (91.4)	*Ref.*		*Ref.*		*Ref.*		*Ref.*	
Black	114 (2.0)	0.61 (0.33, 1.14)	0.123	0.42 (0.22, 0.82)	**0.012**	1.72 (1.02, 2.89)	**0.040**	1.26 (0.73, 2.15)	0.406
Asian	106 (1.9)	2.05 (1.32, 3.18)	**0.001**	1.82 (1.13, 2.93)	**0.014**	1.30 (0.79, 2.12)	0.297	1.10 (0.66, 1.83)	0.726
Hispanic	269 (4.7)	1.03 (0.73, 1.45)	0.874	1.20 (0.81, 1.77)	0.365	1.29 (0.94, 1.76)	0.109	1.07 (0.75, 1.52)	0.710
**NSAID Users**									
White	2079 (93.7)	*Ref.*		*Ref.*		*Ref.*		*Ref.*	
Black	66 (3.0)	1.25 (0.68, 2.33)	0.472	1.05 (0.55, 1.98)	0.887	0.94 (0.52, 1.69)	0.841	0.91 (0.48, 1.71)	0.766
Asian	25 (1.1)	1.62 (0.64, 4.07)	0.310	1.46 (0.57, 3.77)	0.429	0.88 (0.35, 2.21)	0.781	0.81 (0.32, 2.07)	0.660
Hispanic	50 (2.3)	0.44 (0.16, 1.24)	0.123	0.37 (0.11, 1.24)	0.109	1.26 (0.61, 2.62)	0.532	1.01 (0.47, 2.17)	0.985

The interaction between each race and NSAID use were all $p \geq 0.072$ for acute inflammation and $p \geq 0.348$ for chronic inflammation

Abbreviations: 95% CI, 95% confidence interval; OR odds ratio.

*Adjusted for baseline age, race, region, DRE (digital rectal examination), prostate volume, PSA (prostate-specific antigen), smoking, aspirin/NSAID use, and biopsy results.
